# Fragile X mental retardation protein and synaptic plasticity

**DOI:** 10.1186/1756-6606-6-15

**Published:** 2013-04-08

**Authors:** Michael S Sidorov, Benjamin D Auerbach, Mark F Bear

**Affiliations:** 1 The Picower Institute for Learning and Memory, Massachusetts Institute of Technology, 77 Massachusetts Avenue, Cambridge, MA, 02139 46-3301, USA

**Keywords:** FMRP, Protein synthesis, Synaptic plasticity, Long-term depression, Long-term potentiation, Metabotropic glutamate receptor, Fragile X

## Abstract

Loss of the translational repressor FMRP causes Fragile X syndrome. In healthy neurons, FMRP modulates the local translation of numerous synaptic proteins. Synthesis of these proteins is required for the maintenance and regulation of long-lasting changes in synaptic strength. In this role as a translational inhibitor, FMRP exerts profound effects on synaptic plasticity.

## Background

The long-term maintenance of many forms of synaptic plasticity requires the synthesis of new proteins. While the role of experience-dependent somatic gene transcription in long-term memory has been well studied [1], many mRNAs are trafficked to dendrites suggesting an additional role for local synaptic control of protein synthesis [2]. Indeed, activity-dependent translation of pre-existing dendritic mRNA at the synapse is necessary for the expression of multiple forms of synaptic plasticity [3-5]. Fragile X mental retardation protein (FMRP) influences this synaptic plasticity by functioning as a key regulator of mRNA translation [6-10].

FMRP was first characterized in the context of Fragile X syndrome. The *FMR1* gene is silenced in Fragile X (FX), and the consequent loss of FMRP leads to the symptoms of the disorder, often including intellectual disability and autism. In the *Fmr1* KO mouse model [11], loss of FMRP results in increased levels of protein synthesis [9,12]. The downstream consequences of this increase are believed to at the core of FX pathophysiology [13-15]. Rapid progress has been made characterizing how loss of FMRP influences synaptic function and plasticity, and this knowledge has led to several strategies to correct the disorder that have been validated in animals and are now being tested in humans [16-19].

Here we briefly review the evidence, mostly from the *Fmr1* KO mouse, suggesting a role for FMRP in synaptic plasticity. Although the distinction is not always clear-cut, it is conceptually important to separate disruptions of synaptic plasticity that are *consequences* of altered brain development from those disruptions of synaptic plasticity that *cause* altered brain function in the *Fmr1* KO. While both are important for understanding disease pathophysiology, only the latter is relevant to the question of how FMRP contributes to synaptic plasticity in the wild-type brain.

### FMRP regulates translation

FMRP is an RNA-binding protein and a repressor of translation which is well-conserved from mouse to human. FMRP associates with mRNAs through one of three RNA-binding domains [20,21], in some cases in conjunction with adaptor proteins [22,23]. There is evidence that FMRP can repress translation both by blocking initiation and elongation [15,24,25]. A point mutation in one FMRP/mRNA binding domain is sufficient to recapitulate plasticity phenotypes seen in the *Fmr1* KO mouse [26] and in at least one case FX in a human patient [27]. Thus it is likely that FMRP regulates plasticity mainly in its role as a repressor of translation.

FMRP is regulated by posttranslational modifications. Phosphorylated FMRP stalls ribosomal translocation and inhibits translation, whereas dephosphorylation of FMRP upregulates translation [28-30]. Bidirectional regulation of FMRP phosphorylation by the S6 kinase and protein phosphatase 2A (PP2A) in response to activity provide a potential link between synaptic stimulation and local translation [24].

### FMRP is well-positioned to regulate synaptic plasticity

FMRP is well-positioned to be a key regulator of synaptic plasticity for three main reasons. First, the protein is found in dendritic spines [31-34], important postsynaptic sites of plasticity induction and maintenance. Secondly, FMRP regulates dendritic mRNA translation [16,17], which is required for multiple forms of plasticity [35]. Finally, FMRP itself is dynamically regulated by activity: experience and synaptic activation can trigger its local translation and rapid degradation, in addition to the posttranslational regulation mentioned above. Multiple experimental manipulations associated with synaptic plasticity have been shown to increase FMRP levels, including exposure to an enriched environment, a complex learning task, and pharmacological activation of group 1 metabotropic glutatmate receptors (mGluRs) [31,36-38]. Importantly, FMRP is synthesized rapidly, on the same time scale (10–30 minutes) as induction of stable synaptic plasticity [37]. In hippocampal cultures, activity- and mGluR-dependent increases in dendritic FMRP may result from increased trafficking of existing FMRP, rather than *de novo* FMRP synthesis [33,39,40]. Either way, FMRP is an ideal candidate to be involved in regulating synaptic plasticity because of its rapid, transient rise in dendrites following well-characterized plasticity induction paradigms, as well as its role as an inhibitor of translation.

### FMRP regulates mGluR-LTD via protein synthesis

Long-term potentiation (LTP) and long-term depression (LTD) are well-characterized forms of synaptic plasticity associated with learning and memory. These persistent changes in synaptic strength can be induced by a variety of manipulations and their expression mechanisms are diverse. Different induction protocols rely on different mechanisms for maintenance, including the requirement for protein synthesis. A particularly compelling example of a form of plasticity requiring local translation is metabotropic glutamate receptor-dependent LTD (mGluR-LTD) in the CA1 region of the hippocampus. Activation of group 1 mGluRs (mGluR1 and 5), either with paired-pulse low-frequency synaptic stimulation (PP-LFS) [4] or with the selective agonist (*S*)-3,5-dihydroxyphenylglycine (DHPG) [41-43], results in a persistent decrease in synaptic strength that is mechanistically distinct from classical NMDA receptor (NMDAR)-dependent LTD [41,44]. It is important to note that there are several mechanisms downstream of mGluR activation that can depress synaptic transmission, and these can be differentially expressed depending on the induction protocol, age, rearing history, and species (*e.g.*, [44-48]). However, under appropriate experimental conditions the maintenance of mGluR-LTD requires rapid protein synthesis within minutes of induction [4,49]. This protein synthesis is likely to be synaptic, as mGluR-LTD can still be induced if the dendritic layer is physically severed from the cell body layer [4]. mGluR-LTD is expressed, in part, by the removal of AMPA receptors from synapses, which also requires rapid *de novo* translation [50]. The new protein synthesis may be instructive rather than merely permissive for synaptic plasticity since activation of group 1 mGluRs rapidly stimulates protein synthesis in hippocampal slices [12], dendrites and synaptoneurosomes [51,52].

**Figure 1:**
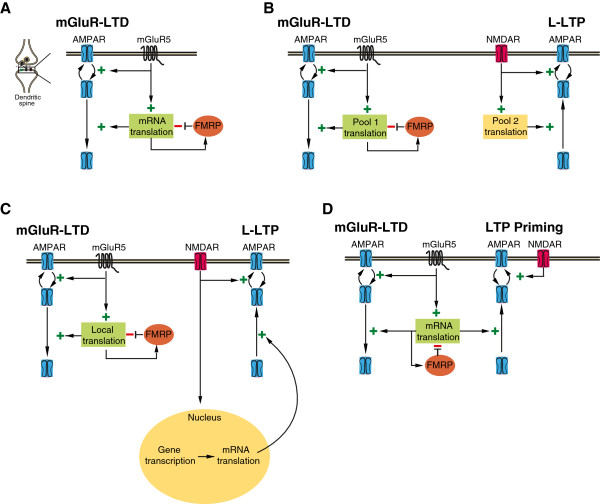
**The role of FMRP in translation-dependent synaptic plasticity.** (**A**) FMRP and mGluR5 impose opposite regulation on the local mRNA translation required for mGluR-LTD expression. In the absence of FMRP, there is excessive protein synthesis and exaggerated LTD. (**B**) While FMRP is known to regulate the translation required for LTD, evidence suggests it is not involved in the expression of L-LTP. There may be different pools of mRNA available at the synapse that are differentially required for LTD versus LTP, and FMRP may specifically regulate the pool required for LTD. (**C**) FMRP is explicitly involved in the regulation of dendritically localized translation and may not regulate somatic translation. Consequently, FMRP may only impact forms of plasticity that require local translation, such as mGluR-LTD. (**D**) In addition to mGluR-LTD, FMRP regulates the protein synthesis involved in mGluR-dependent facilitation of LTP. This finding suggests that the proteins whose translation is controlled by FMRP may be involved in bi-directional maintenance of plasticity rather than being specific to LTD.

*Fmr1* knockout mice show enhanced hippocampal mGluR-LTD [8,14,49,53] (Table 1). A subsequent study found a similar enhancement in cerebellar mGluR-LTD, which shares many of the same expression mechanisms [54]. Consistent with the electophysiological data, loss of FMRP leads to excessive mGluR-mediated AMPAR internalization [55]. In addition, mGluR-LTD no longer requires new protein synthesis in the *Fmr1* KO mice [49,56]. These results, combined with what is known about FMRP function, suggest that FMRP acts to inhibit the synthesis of proteins required for mGluR-LTD. In the absence of FMRP, these “LTD proteins” are already available or over-expressed in dendrites resulting in enhanced magnitude and protein synthesis-independent persistence of this form of plasticity (Figure 1A) [13]. Conversely, postnatal overexpression of FMRP reduces the magnitude of mGluR-LTD in both wildtype and *Fmr1* KO neurons [49] and restores its protein synthesis dependence [57]. Moreover, reducing mGluR5 signaling in *Fmr1* KO mice restores both protein synthesis rates and LTD magnitude in the hippocampus to wildtype levels [53,58], suggesting that mGluR5 and FMRP act in functional opposition to maintain an optimal level of synaptic protein synthesis throughout development and into adulthood (Figure 1A).

**Table 1:** Fragile X mouse synaptic plasticity phenotypes

**Category**	**Region**	**Fragile X Mouse Phenotype**	**Age**	**References**
mGluR LTD	hippocampus	enhanced	P25-30	Huber et al., 2002; Hou et al., 2006; Bhattacharya et al., 2012; Michalon et al., 2012
mGluR LTD	hippocampus	does not require new protein synthesis	4-12 wk	Nosyreva and Huber, 2006; Hou, et al., 2006; Zang et al., 2009**
mGluR LTD	hippocampus	*enhanced and not PS-dependent	P35-42	Iliff et al., 2012
mGluR LTD	cerebellum	enhanced	3-7 wk	Koekkoek et al., 2005
mGluR LTD	hippocampus	enhanced and does not require new protein synthesis	3-7 wk	Volk et al., J Neurosci, 2007
LTP	hippocampus	NONE	20-26 wk; 8-10 wk; 3-12 month	Godfraind et al., 1996; Li et al., 2002; Larson et al., 2005
L-LTP	hippocampus	NONE	5-7 wk; 2-4 month	Paradee et al., 1999; Zhang et al., *J* 2009
LTP	hippocampus	deficient	2 wk; 6-8 wk	Hu et al., 2008; Shang et al., 2009
LTP	hippocampus	deficient with weak stimulus; normal with strong stimulus	2-3 month	Lauterborn et al., 2007
LTP	hippocampus	enhanced B-adrenergic-facilitated heterosynaptic LTP (PS-dependent)	3-4 month	Connor et al., 2011
LTP priming	hippocampus	does not require new protein synthesis (mGluR-dependent)	6-10 wk	Auerbach and Bear, 2010
LTP	anterior cingulate ctx	deficient	6-8 wk	Zhao et al., 2005; Xu et al., 2012
LTP	anterior cingulate ctx	impaired facilitation of LTP by 5-HT2AR agonist	6-8 wk	Xu et al., 2012
LTP	somatosensory, temporal ctx	deficient	8-10 wk; 3 month	Li et al., 2002; Hayashi et al., 2007
LTP	somatosensory ctx	delayed window for plasticity	P3-10	Harlow et al., 2010
LTP	visual ctx	deficient (mGluR-dependent)	P13-25	Wilson and Cox, 2007
LTP	anterior piriform ctx	deficient in aged mice; normal in 3-6 mo mice	6-18 month	Larson et al., 2005
LTP	amygdala	impaired (mGluR-dependent)	6-8 wk; 3.5-6 mo	Zhao et al., 2005; Suvrathan et al., 2010
STD-LTP	somatosensory ctx	deficient with weak stimulus	P10-18	Desai et al., 2006
STD-LTP	prefrontal ctx	deficient with weak stimulus; normal with strong stimulus	P14-23	Meredith et al., 2007
homeostasis	hippocampus	deficient translation-dependent scaling	P6-7 slice culture	Soden and Chen, 2010
homeostasis	hippocampus	normal transcription-dependent scaling	P6-7 slice culture	Soden and Chen, 2010
experience-dependent	visual ctx (in vivo)	altered ocular dominance plasticity	LTD	Dolen et al., 2007
experience-dependent	somatosensory ctx	deficient experience-dependent plasticity (induced by whisker trimming)	LTD	Bureau et al., 2008

LEGEND: Fragile X mouse models have multiple altered forms of synaptic plasticity across multiple brain regions. The majority of phenotypes were assessed in the *Fmr1* KO mouse which lacks FMRP. *Assessed using a CGG knock-in mouse which models FX premutation. **Assessed using an FMRP point mutant mouse with disrupted FMRP-mRNA binding.

### L-LTP appears normal in *Fmr1* KO mice

While the effects of protein synthesis inhibition on mGluR-LTD can be seen within minutes, most forms of synaptic plasticity do not require *de novo* synthesis until several hours after induction. This is best characterized by late phase LTP (L-LTP), a persistent form of potentiation lasting at least 4 hours. The late maintenance phase of L-LTP requires protein synthesis but initial induction does not [59,60]. Due to FMRP’s conjectured role in translation regulation, L-LTP was one of the first forms of plasticity studied in the *Fmr1* KO mouse [61]. Interestingly, no difference has been found in the magnitude of L-LTP in the *Fmr1* KO [61,62]. The fact that removal of FMRP affects protein synthesis-dependent LTD but not LTP suggests that FMRP may specifically regulate the translation of proteins required for the expression of LTD (Figure 1B). However, while the magnitude of L-LTP is unchanged, it is possible that L-LTP is qualitatively different in its requirement for new protein synthesis when FMRP is absent, as is the case for mGluR-LTD (and LTP priming, see below). Therefore, it will be important to test the protein synthesis-dependency of L-LTP in *Fmr1* KO mice to show that FMRP truly does not play a role in regulating the persistence of LTP.

Alternatively, FMRP may be required for the regulation of local but not somatic translation in the context of L-LTP (Figure 1C). L-LTP is traditionally induced by multiple trains of high frequency tetanus or theta burst stimulation, protocols that rely on cell-wide transcription and translation [63-65]. L-LTP was characterized in the *Fmr1* KO mouse using these classical paradigms [61,62]. However, using a less intense induction protocol results in L-LTP that is maintained specifically by local dendritic translation [5,66]. This form of L-LTP, similar to mGluR-LTD, is sensitive to inhibitors of translation but not transcription, and can be maintained in isolated dendrites. It will be interesting to determine if this locally expressed form of L-LTP is regulated by FMRP.

### FMRP regulates LTP priming

While the role of FMRP in L-LTP is unclear, FMRP is known to be involved in LTP in other contexts. In particular, FMRP is involved in regulation of an mGluR-dependent form of metaplasticity that sets the threshold for LTP. Originally described in rats [67], weak activation of group 1 mGluRs, in itself insufficient for LTD induction, facilitates the subsequent induction of LTP (“LTP priming”). As with mGluR-LTD, this facilitation requires translation but not transcription [68]. This prompted the examination of the role of FMRP in LTP priming [69]. mGluR-dependent priming of LTP is of comparable magnitude in WT and *Fmr1* KO mice; however, while LTP priming requires acute stimulation of protein synthesis in WT mice, it is no longer protein synthesis-dependent in the *Fmr1* KO. Thus, while mGluR-LTD and LTP priming are qualitatively different functional consequences of Gp1 mGluR-stimulated protein synthesis in the hippocampus, both processes are altered by the removal of FMRP (Figure 1D). These results suggest that the mRNA under translational control of FMRP may code for proteins required for bidirectional changes in synaptic strength. Thus, the proteins regulated by FMRP should be conceptualized as plasticity gatekeepers rather than solely “LTD proteins.”

### The induction threshold for LTP and STD-LTP is raised in *Fmr1* KO mice

**Figure 2:**
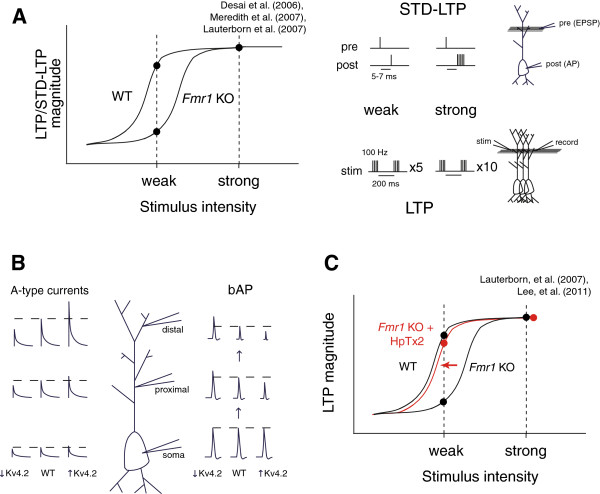
**FMRP and Kv4.2 regulate the threshold for inducing synaptic potentiation.** (**A**) FMRP sets the threshold for LTP and STD-LTP. *Fmr1* KO mice have deficient hippocampal LTP and cortical STD-LTP only with a “weak” induction protocol. (**B**) Kv4.2 is a dendritic A-type K+ channel that attenuates action potential backpropagation (bAP) and dendritic excitability. (**C**) Inhibition of Kv4.2 restores LTP following a weak induction protocol in *Fmr1* KO mice.

In *Fmr1* KO hippocampal slices, LTP induction is deficient with a weak 5 theta burst protocol but is normal with a strong 10 theta burst protocol (Figure 2A) [70]. In addition, FMRP modulates the induction threshold for spike-timing dependent long-term potentiation (STD-LTP). This form of Hebbian plasticity is induced by temporally staggered presynaptic and postsynaptic activity within a very short window [71,72]. In somatosensory and prefrontal cortices, STD-LTP is deficient in *Fmr1* KO neurons [73,74]. However, if the postsynaptic stimulus strength is increased from a single spike to a burst of five spikes, STD-LTP does occur in KO neurons (Figure 2A) [74]. Therefore FMRP is not required for expression of STD-LTP, but the threshold is raised in its absence. A possible mechanism for ongoing regulation of LTP thresholds by FMRP is discussed later in this review.

### FMRP and other translation-dependent forms of plasticity

In addition to its role in translation-dependent forms of Hebbian plasticity, FMRP can also modulate some forms of homeostatic plasticity. Synaptic scaling is a form of homeostatic plasticity that acts to keep the strength of synapses within a functional range in response to extreme changes in activity. Broadly, a decrease in activity leads to a subsequent cell-wide increase in synaptic strength (“scaling up”) and an increase in activity leads to a decrement in synaptic strength (“scaling down”) [75]. Two types of scaling up have been described in hippocampal slice culture: one that requires transcription [76] and one that requires local translation [77]. Interestingly, only the translation-dependent form of synaptic scaling is deficient in neurons lacking FMRP. Postsynaptic viral expression of FMRP corrects deficient translation-dependent scaling up in *Fmr1* KO neurons [78]. Scaling down of synapses in response to high levels of activity (following prolonged blockade of inhibition) has also been observed [79] and requires mGluR5 activation [80,81]. However, the role of FMRP and local protein synthesis in scaling down has not been directly examined.

While the role of FMRP has been best characterized in mGluR-dependent forms of plasticity, it is not specific to these receptors. Removal of FMRP occludes TrkB-mediated increases in protein synthesis [12] and alters other forms of G protein-coupled receptor (GPCR)-dependent LTD and LTP [82,83]. The common thread between these processes is their reliance on local dendritic translation. Indeed, evidence suggests that FMRP may specifically be important for the regulation of local rather than somatic translation (Figure 1C), as removal of FMRP affects translation but not transcription-dependent forms of Hebbian and homeostatic plasticity.

### FMRP and translation-independent plasticity

While many forms of translation-dependent synaptic plasticity are abnormal in *Fmr1* KO mice, other forms of hippocampal plasticity, including NMDAR-dependent LTD and early-phase LTP, are normal [8,61,69,84,85]. These observations suggest that FMRP regulates plasticity mainly in its role as a regulator of translation. However, removal of FMRP has also been shown to affect some forms of synaptic plasticity that do not require *de novo* translation, such as early-phase LTP in other brain areas, including the cortex and amygdala [61,85-89]. Some of these effects could be explained by FMRP modulation of protein synthesis-dependent plasticity thresholds; however it seems likely that many represent end-stage consequences of altered synaptic development in the *Fmr1* KO.

A case in point is altered LTP in the amygdala. A substantial deficit in basal transmission was reported at the same synapses that showed impaired LTP [88]. Reduced synaptic connectivity might have caused the defective LTP, and might have arisen as a consequence of increased FMRP-dependent protein synthesis during the development of amygdala circuitry.

### Candidate plasticity gating proteins regulated by FMRP

In order to determine how FMRP regulates synaptic plasticity, we must identify the synaptic proteins whose translation is regulated by FMRP. FMRP has a wide variety of targets - it has been shown to selectively bind approximately 4% of the mRNA in the mammalian brain [90]. Recently, over 800 mRNA binding targets of FMRP were identified using a novel high throughput cross-linking immunoprecipitation (HITS-CLIP) assay [10]. These targets include genes coding for pre- and post-synaptically expressed proteins: 27% of pre-synaptic protein mRNAs (90 genes) and 23% of postsynaptic protein mRNAs (257 genes) are FMRP targets [10]. More specifically, the HITS-CLIP study found that 31% of mRNAs coding for proteins in the NMDAR complex (58 genes), 62% in the mGluR5 complex (32 genes), and 33% in the AMPAR complex (3 genes) are FMRP targets. These three receptor complexes are important for the induction and maintenance of synaptic plasticity, suggesting that FMRP likely acts broadly as a translational regulator rather than solely regulating one or two “plasticity proteins.”

The finding that many FMRP targets encode presynaptic proteins is interesting and illuminating. In the mature nervous system the evidence for local protein synthesis in axons or axon terminals is still sparse; however during early axon development and synapse formation local protein synthesis is believed to play an important role in pathway and target selection [91,92]. Thus, the absence of FMRP regulation of protein synthesis during early development very likely alters synaptic connectivity well before the onset of experience-dependent postnatal plasticity. In addition, outside the CNS, local control of translation in sensory afferent terminals plays a role in nociceptive sensitization and neuropathic pain [93]. FMRP is localized to these terminals and *Fmr1* KO mice show altered nociceptive sensitization [94]. These results suggest that in the spinal cord, presynaptic FMRP may inhibit local translation and can regulate pain plasticity even into adulthood.

We have discussed two major categories of plasticity defects in *Fmr1* KO mice: (1) forms of plasticity requiring FMRP/local translation for their maintenance (mGluR-LTD) and (2) forms of plasticity where FMRP regulates their induction threshold (STD-LTP). We will discuss a few proteins in both categories that are likely involved given their regulation by FMRP and their known roles in plasticity maintenance and threshold-setting in wild-type synapses. These “candidate proteins” are meant to serve as exemplars of how FMRP might regulate synaptic plasticity.

#### Plasticity maintenance proteins: MAP1B, Arc, and STEP

Recent work has identified proteins whose translation is regulated by FMRP and are involved in mGluR-LTD, including microtubule-associated protein 1B (MAP1B) and activity-regulated cytoskeleton-associated protein (Arc) [17,18]. MAP1B is required for mGluR-depdendent AMPA receptor endocytosis [95], the mechanism by which mGluR-LTD is expressed. FMRP associates with MAP1B mRNA and represses its translation [90,96-98], and *Fmr1* KO mice show increased hippocampal MAP1B expression [49]. However, there may be mouse strain and region-specific variations in how FMRP regulates MAP1B translation. For example, in the cerebellum and hippocampus of FVB mice, FMRP may positively regulate MAP1B expression [99].

Arc is involved in AMPAR endocytosis [100] and is upregulated in dendrites following mGluR activation [101,102] and behavior [103]. Arc is required for hippocampal mGluR-LTD and L-LTP, which are both protein synthesis-dependent, and Arc^−/−^ mice have multiple learning deficits [101,102,104]. FMRP binds Arc mRNA and suppresses its translation. As a result, Arc expression is increased in *Fmr1* KO dendrites [98,105,106]. Since (a) mGluR-LTD is increased in *Fmr1* KO mice, (b) Arc is increased in *Fmr1* KO dendrites, and (c) Arc is required for mGluR-LTD, it seems likely that FMRP regulates mGluR-LTD via Arc. This hypothesis was tested directly using Fmr1/Arc double knockout mice which show deficient (rather than exaggerated) mGluR-LTD [8,102]. This finding suggests that increased Arc expression may partially account for the enhanced mGluR-LTD seen in *Fmr1* KO mice.

Mechanistically, dephosphorylation of FMRP by the phosphatase PP2A is required for rapid mGluR-mediated increases in Arc protein. However in *Fmr1* KO neurons, Arc levels are basally increased, occluding a further effect of DHPG treatment. Acute viral reintroduction of FMRP into *Fmr1* KO neurons normalizes dendritic Arc levels and restores rapid mGluR-mediated Arc synthesis. This provides further evidence that the acute loss of FMRP, rather than developmental abnormality, underlies synaptic plasticity phenotypes in the *Fmr1* knockout mouse. eregulation of translation.

In addition to MAP1B and Arc, numerous other candidate LTD proteins have been identified in the *Fmr1* KO mouse. One interesting example is striatal-enriched protein tyrosine phosphatase (STEP). Translation of STEP is increased during mGluR-LTD [107,108], and STEP mRNA binds to FMRP [10]. Genetic reduction of STEP corrects behavioral phenotypes in the *Fmr1* KO mouse; but it is not known whether corresponding LTD phenotypes are affected [109]. Additional candidate proteins include APP [110,111], OPHN1 [112], CaMKIIα [49,98,113], PSD-95 [113-115], and PI3K [116].

#### Plasticity threshold-regulating proteins: Kv4.2

A recent review discussing the role of potassium channels in Fragile X provides insight into how FMRP may regulate excitability [117]. FMRP directly regulates the translation of at least three potassium channels: Kv4.2, Kv3.1b, and Slack [118-122]. FMRP’s control of Kv4.2 translation may have indirect consequences on regulating the threshold for LTP and STD-LTP induction.

Kv4.2 is an A-type potassium channel that regulates dendritic excitability and the extent of action potential backpropagation [123,124]. A-type currents act to dampen dendritic excitability and AP backpropagation (Figure 2B). By modulating the strength of backpropagation, Kv4.2 also has been shown to regulate the threshold for LTP and STD-LTP [123,125]. In the absence of Kv4.2, dendrites are more excitable and there is a decreased threshold for LTP induction [123,126].

*Fmr1* KO mice have an increased threshold for LTP and STD-LTP induction, as discussed earlier (Figure 2A) [73,74]. One potential hypothesis for this phenomenon is that FMRP inhibits the translation of Kv4.2, and *Fmr1* KO mice have excessive Kv4.2 protein synthesized in dendrites. Indeed, FMRP does directly associate with and negatively regulate the translation of Kv4.2 mRNA [118]. But does this account for the altered LTP/STD-LTP threshold in *Fmr1* KO mice? Pharmacological inhibition of Kv4.2 in *Fmr1* KO mice does correct deficient weak-stimulus hippocampal LTP while strong-stimulus LTP remains unchanged [118] (Figure 2C). This finding suggests that the increased threshold for LTP in the *Fmr1* KO mouse may be accounted for by increased translation of the potassium channel Kv4.2.

Interestingly, another group has recently shown that under their conditions, FMRP positively regulates the translation of Kv4.2 [119]. This study did not address the potential consequences of decreased Kv4.2 in the *Fmr1* KO on synaptic plasticity. One would expect increased dendritic excitability, which has been previously reported in other contexts [127], and a decreased LTP threshold. It will be important to determine the precise experimental and *in vivo* conditions under which each of these opposing patterns of regulation can occur, but it is clear that FMRP’s regulation of Kv4.2 in either direction would have important consequences for plasticity.

### FMRP, synaptic plasticity and learning

Long-lasting synaptic potentiation and depression have long been considered potential neural correlates of learning and memory. In conjunction with FMRP’s role in synaptic plasticity in multiple brain areas, FMRP is also important for a wide range of behavioral learning tasks in mice. *Fmr1* KO mice show deficient amygdalar trace fear memory [87], cerebellar learning [54], inhibitory avoidance learning [58], and have difficulties with a prefrontal cognitive learning task [128]. Drosophila mutants lacking FMRP also have impaired long-term memory [129]. Overall, learning and memory deficits in the *Fmr1* KO mouse are a likely behavioral consequence of abnormal synaptic plasticity.

## Conclusions

FMRP participates in the regulation of numerous forms of synaptic plasticity, including mGluR-LTD, LTP priming, and synaptic scaling. It seems that FMRP is particularly important for synaptic plasticity that requires dendritic translation, as these forms of plasticity all require local translation and FMRP is a well-established regulator of local translation. The current evidence suggests that FMRP plays an essential role in regulating the synaptic expression of proteins required for bidirectional changes in synaptic strength (Figure 1). It is likely that FMRP controls the expression of proteins not only acutely required for expression of synaptic plasticity, but also proteins that regulate the threshold for plasticity induction (Figure 2). Therefore FMRP’s role in synaptic plasticity is two-fold: it regulates the translation of proteins that directly participate in the induction and expression of plasticity as well as proteins that can indirectly modulate the properties of plasticity.

A key goal in the Fragile X field is to identify which proteins are regulated by FMRP and how increases or decreases in these proteins may account for phenotypes of the disorder. Determining the proteins that are regulated by FMRP (and altered in FX) will also lead to a better understanding of the neuronal processes that are essential for synaptic plasticity and learning/memory. The HITS-CLIP screen has identified hundreds of candidates and a significant number of these are putatively involved in synaptic plasticity. It is unlikely that there is one global “plasticity protein” - multiple proteins likely regulate different processes in parallel. Mapping which proteins are essential for which processes is the important next step for understanding the role of FMRP in the pathological and non-pathological brain.

The *Fmr1* KO mouse provides a model for assessing the role of FMRP in synaptic plasticity - but on their own, studies in *Fmr1* KO mice leave open the possibility that developmental rather than acute changes result in altered synaptic plasticity. In multiple contexts, acute manipulations of FMRP suggest that FMRP does actively regulate synaptic plasticity as a regulator of translation. There is ample evidence that FMRP can directly impact synaptic plasticity through its control of protein synthesis. Future work that allows for better temporal and spatial control of FMRP expression will help dissect the role of FMRP in development from its acute effects on synaptic plasticity.

## Competing interests

The authors declare that they have no competing interests.

## Authors’ contributions

All authors drafted, read, and approved the manuscript.

## References

[B1] Kandel ER The molecular biology of memory storage: a dialogue between genes and synapses Science 2001 294 1030 1038 10.1126/science.1067020 11691980

[B2] Steward O Levy WB Preferential localization of polyribosomes under the base of dendritic spines in granule cells of the dentate gyrus J Neurosci 1982 2 284 291 7062109 10.1523/JNEUROSCI.02-03-00284.1982 PMC6564334

[B3] Kang H Schuman EM A requirement for local protein synthesis in neurotrophin-induced hippocampal synaptic plasticity Science 1996 273 1402 1406 10.1126/science.273.5280.1402 8703078

[B4] Huber KM Kayser MS Bear MF Role for rapid dendritic protein synthesis in hippocampal mGluR-dependent long-term depression Science 2000 288 1254 1257 10.1126/science.288.5469.1254 10818003

[B5] Huang YY Kandel ER Theta frequency stimulation induces a local form of late phase LTP in the CA1 region of the hippocampus Learn Mem 2005 12 587 593 10.1101/lm.98905 16287724 PMC1356176

[B6] Laggerbauer B Ostareck D Keidel EM Ostareck-Lederer A Fischer U Evidence that fragile X mental retardation protein is a negative regulator of translation Hum Mol Genet 2001 10 329 338 10.1093/hmg/10.4.329 11157796

[B7] Li Z Zhang Y Ku L Wilkinson KD Warren ST Feng Y The fragile X mental retardation protein inhibits translation via interacting with mRNA Nucleic Acids Res 2001 29 2276 2283 10.1093/nar/29.11.2276 11376146 PMC55699

[B8] Huber KM Gallagher SM Warren ST Bear MF Altered synaptic plasticity in a mouse model of fragile X mental retardation Proc Natl Acad Sci USA 2002 99 7746 7750 10.1073/pnas.122205699 12032354 PMC124340

[B9] Qin M Kang J Burlin TV Jiang C Smith CB Postadolescent changes in regional cerebral protein synthesis: an in vivo study in the FMR1 null mouse J Neurosci 2005 25 5087 5095 10.1523/JNEUROSCI.0093-05.2005 15901791 PMC6724856

[B10] Darnell JC Van Driesche SJ Zhang C Hung KY Mele A Fraser CE Stone EF Chen C Fak JJ Chi SW FMRP stalls ribosomal translocation on mRNAs linked to synaptic function and autism Cell 2011 146 247 261 10.1016/j.cell.2011.06.013 21784246 PMC3232425

[B11] Consortium TD-BFX Fmr1 knockout mice: a model to study fragile X mental retardation Cell 1994 78 23 33 8033209

[B12] Osterweil EK Krueger DD Reinhold K Bear MF Hypersensitivity to mGluR5 and ERK1/2 leads to excessive protein synthesis in the hippocampus of a mouse model of fragile X syndrome J Neurosci 2010 30 15616 15627 10.1523/JNEUROSCI.3888-10.2010 21084617 PMC3400430

[B13] Bear MF Huber KM Warren ST The mGluR theory of fragile X mental retardation Trends Neurosci 2004 27 370 377 10.1016/j.tins.2004.04.009 15219735

[B14] Bhattacharya A Kaphzan H Alvarez-Dieppa AC Murphy JP Pierre P Klann E Genetic removal of p70 S6 kinase 1 corrects molecular, synaptic, and behavioral phenotypes in fragile X syndrome mice Neuron 2012 76 325 337 10.1016/j.neuron.2012.07.022 23083736 PMC3479445

[B15] Bhakar AL Dolen G Bear MF The pathophysiology of fragile X (and what it teaches us about synapses) Annu Rev Neurosci 2012 35 417 443 10.1146/annurev-neuro-060909-153138 22483044 PMC4327822

[B16] Garber K Smith KT Reines D Warren ST Transcription, translation and fragile X syndrome Curr Opin Genet Dev 2006 16 270 275 10.1016/j.gde.2006.04.010 16647847

[B17] Bassell GJ Warren ST Fragile X syndrome: loss of local mRNA regulation alters synaptic development and function Neuron 2008 60 201 214 10.1016/j.neuron.2008.10.004 18957214 PMC3691995

[B18] Pfeiffer BE Huber KM The state of synapses in fragile X syndrome Neuroscientist 2009 15 549 567 10.1177/1073858409333075 19325170 PMC2762019

[B19] Krueger DD Bear MF Toward fulfilling the promise of molecular medicine in fragile X syndrome Annu Rev Med 2011 62 411 429 10.1146/annurev-med-061109-134644 21090964 PMC3100156

[B20] Ashley CT Jr Wilkinson KD Reines D Warren ST FMR1 protein: conserved RNP family domains and selective RNA binding Science 1993 262 563 566 10.1126/science.7692601 7692601

[B21] Siomi H Siomi MC Nussbaum RL Dreyfuss G The protein product of the fragile X gene, FMR1, has characteristics of an RNA-binding protein Cell 1993 74 291 298 10.1016/0092-8674(93)90420-U 7688265

[B22] Napoli I Mercaldo V Boyl PP Eleuteri B Zalfa F De Rubeis S Di Marino D Mohr E Massimi M Falconi M The fragile X syndrome protein represses activity-dependent translation through CYFIP1, a new 4E-BP Cell 2008 134 1042 1054 10.1016/j.cell.2008.07.031 18805096

[B23] El Fatimy R Tremblay S Dury AY Solomon S De Koninck P Schrader JW Khandjian EW Fragile X mental retardation protein interacts with the RNA-binding protein Caprin1 in neuronal RiboNucleoProtein complexes [corrected] PLoS One 2012 7 e39338 10.1371/journal.pone.0039338 22737234 PMC3380850

[B24] Santoro MR Bray SM Warren ST Molecular mechanisms of fragile X syndrome: a twenty-year perspective Annu Rev Pathol 2012 7 219 245 10.1146/annurev-pathol-011811-132457 22017584

[B25] Bagni C Greenough WT From mRNP trafficking to spine dysmorphogenesis: the roots of fragile X syndrome Nat Rev Neurosci 2005 6 376 387 15861180 10.1038/nrn1667

[B26] Zang JB Nosyreva ED Spencer CM Volk LJ Musunuru K Zhong R Stone EF Yuva-Paylor LA Huber KM Paylor R A mouse model of the human Fragile X syndrome I304N mutation PLoS Genet 2009 5 e1000758 10.1371/journal.pgen.1000758 20011099 PMC2779495

[B27] De Boulle K Verkerk AJ Reyniers E Vits L Hendrickx J Van Roy B Van den Bos F de Graaff E Oostra BA Willems PJ A point mutation in the FMR-1 gene associated with fragile X mental retardation Nat Genet 1993 3 31 35 10.1038/ng0193-31 8490650

[B28] Coffee RL Jr Williamson AJ Adkins CM Gray MC Page TL Broadie K In vivo neuronal function of the fragile X mental retardation protein is regulated by phosphorylation Hum Mol Genet 2012 21 900 915 10.1093/hmg/ddr527 22080836 PMC3263990

[B29] Muddashetty RS Nalavadi VC Gross C Yao X Xing L Laur O Warren ST Bassell GJ Reversible inhibition of PSD-95 mRNA translation by miR-125a, FMRP phosphorylation, and mGluR signaling Mol Cell 2011 42 673 688 10.1016/j.molcel.2011.05.006 21658607 PMC3115785

[B30] Ceman S O'Donnell WT Reed M Patton S Pohl J Warren ST Phosphorylation influences the translation state of FMRP-associated polyribosomes Hum Mol Genet 2003 12 3295 3305 10.1093/hmg/ddg350 14570712

[B31] Weiler IJ Irwin SA Klintsova AY Spencer CM Brazelton AD Miyashiro K Comery TA Patel B Eberwine J Greenough WT Fragile X mental retardation protein is translated near synapses in response to neurotransmitter activation Proc Natl Acad Sci USA 1997 94 5395 5400 10.1073/pnas.94.10.5395 9144248 PMC24689

[B32] Feng Y Absher D Eberhart DE Brown V Malter HE Warren ST FMRP associates with polyribosomes as an mRNP, and the I304N mutation of severe fragile X syndrome abolishes this association Mol Cell 1997 1 109 118 10.1016/S1097-2765(00)80012-X 9659908

[B33] Antar LN Afroz R Dictenberg JB Carroll RC Bassell GJ Metabotropic glutamate receptor activation regulates fragile x mental retardation protein and FMR1 mRNA localization differentially in dendrites and at synapses J Neurosci 2004 24 2648 2655 10.1523/JNEUROSCI.0099-04.2004 15028757 PMC6729525

[B34] Ferrari F Mercaldo V Piccoli G Sala C Cannata S Achsel T Bagni C The fragile X mental retardation protein-RNP granules show an mGluR-dependent localization in the post-synaptic spines Mol Cell Neurosci 2007 34 343 354 10.1016/j.mcn.2006.11.015 17254795

[B35] Sutton MA Schuman EM Dendritic protein synthesis, synaptic plasticity, and memory Cell 2006 127 49 58 10.1016/j.cell.2006.09.014 17018276

[B36] Irwin SA Swain RA Christmon CA Chakravarti A Weiler IJ Greenough WT Evidence for altered Fragile-X mental retardation protein expression in response to behavioral stimulation Neurobiol Learn Mem 2000 74 87 93 11001622

[B37] Gabel LA Won S Kawai H McKinney M Tartakoff AM Fallon JR Visual experience regulates transient expression and dendritic localization of fragile X mental retardation protein J Neurosci 2004 24 10579 10583 10.1523/JNEUROSCI.2185-04.2004 15564573 PMC6730125

[B38] Todd PK Malter JS Mack KJ Whisker stimulation-dependent translation of FMRP in the barrel cortex requires activation of type I metabotropic glutamate receptors Brain Res Mol Brain Res 2003 110 267 278 10.1016/S0169-328X(02)00657-5 12591163

[B39] Antar LN Dictenberg JB Plociniak M Afroz R Bassell GJ Localization of FMRP-associated mRNA granules and requirement of microtubules for activity-dependent trafficking in hippocampal neurons Genes Brain Behav 2005 4 350 359 10.1111/j.1601-183X.2005.00128.x 16098134

[B40] Dictenberg JB Swanger SA Antar LN Singer RH Bassell GJ A direct role for FMRP in activity-dependent dendritic mRNA transport links filopodial-spine morphogenesis to fragile X syndrome Dev Cell 2008 14 926 939 10.1016/j.devcel.2008.04.003 18539120 PMC2453222

[B41] Palmer MJ Irving AJ Seabrook GR Jane DE Collingridge GL The group I mGlu receptor agonist DHPG induces a novel form of LTD in the CA1 region of the hippocampus Neuropharmacology 1997 36 1517 1532 10.1016/S0028-3908(97)00181-0 9517422

[B42] Huber KM Bear MF Activation of group 1 metabotropic glutamate receptors induces long-term depression of synaptic transmission in area CA1 of rat hippocampus Soc Neurosci Abstr 1998 22

[B43] Huber KM Roder JC Bear MF Chemical induction of mGluR5- and protein synthesis-dependent long-term depression in hippocampal area CA1 J Neurophysiol 2001 86 321 325 11431513 10.1152/jn.2001.86.1.321

[B44] Oliet SH Malenka RC Nicoll RA Two distinct forms of long-term depression coexist in CA1 hippocampal pyramidal cells Neuron 1997 18 969 982 10.1016/S0896-6273(00)80336-0 9208864

[B45] Zakharenko SS Zablow L Siegelbaum SA Altered presynaptic vesicle release and cycling during mGluR-dependent LTD Neuron 2002 35 1099 1110 10.1016/S0896-6273(02)00898-X 12354399

[B46] Nosyreva ED Huber KM Developmental switch in synaptic mechanisms of hippocampal metabotropic glutamate receptor-dependent long-term depression J Neurosci 2005 25 2992 3001 10.1523/JNEUROSCI.3652-04.2005 15772359 PMC6725134

[B47] Moult PR Correa SA Collingridge GL Fitzjohn SM Bashir ZI Co-activation of p38 mitogen-activated protein kinase and protein tyrosine phosphatase underlies metabotropic glutamate receptor-dependent long-term depression J Physiol 2008 586 2499 2510 10.1113/jphysiol.2008.153122 18356198 PMC2464349

[B48] Auerbach BD Osterweil EK Bear MF Mutations causing syndromic autism define an axis of synaptic pathophysiology Nature 2011 480 63 68 10.1038/nature10658 22113615 PMC3228874

[B49] Hou L Antion MD Hu D Spencer CM Paylor R Klann E Dynamic translational and proteasomal regulation of fragile X mental retardation protein controls mGluR-dependent long-term depression Neuron 2006 51 441 454 10.1016/j.neuron.2006.07.005 16908410

[B50] Snyder EM Philpot BD Huber KM Dong X Fallon JR Bear MF Internalization of ionotropic glutamate receptors in response to mGluR activation Nat Neurosci 2001 4 1079 1085 10.1038/nn746 11687813

[B51] Job C Eberwine J Identification of sites for exponential translation in living dendrites Proc Natl Acad Sci USA 2001 98 13037 13042 10.1073/pnas.231485698 11606784 PMC60820

[B52] Weiler IJ Greenough WT Metabotropic glutamate receptors trigger postsynaptic protein synthesis Proc Natl Acad Sci USA 1993 90 7168 7171 10.1073/pnas.90.15.7168 8102206 PMC47097

[B53] Michalon A Sidorov M Ballard TM Ozmen L Spooren W Wettstein JG Jaeschke G Bear MF Lindemann L Chronic Pharmacological mGlu5 Inhibition Corrects Fragile X in Adult Mice Neuron 2012 74 49 56 10.1016/j.neuron.2012.03.009 22500629 PMC8822597

[B54] Koekkoek SK Yamaguchi K Milojkovic BA Dortland BR Ruigrok TJ Maex R De Graaf W Smit AE VanderWerf F Bakker CE Deletion of FMR1 in Purkinje cells enhances parallel fiber LTD, enlarges spines, and attenuates cerebellar eyelid conditioning in Fragile X syndrome Neuron 2005 47 339 352 10.1016/j.neuron.2005.07.005 16055059

[B55] Nakamoto M Nalavadi V Epstein MP Narayanan U Bassell GJ Warren ST Fragile X mental retardation protein deficiency leads to excessive mGluR5-dependent internalization of AMPA receptors Proc Natl Acad Sci USA 2007 104 15537 15542 10.1073/pnas.0707484104 17881561 PMC2000537

[B56] Nosyreva ED Huber KM Metabotropic receptor-dependent long-term depression persists in the absence of protein synthesis in the mouse model of fragile X syndrome J Neurophysiol 2006 95 3291 3295 10.1152/jn.01316.2005 16452252

[B57] Zeier Z Kumar A Bodhinathan K Feller JA Foster TC Bloom DC Fragile X mental retardation protein replacement restores hippocampal synaptic function in a mouse model of fragile X syndrome Gene Ther 2009 16 1122 1129 10.1038/gt.2009.83 19571888 PMC2741536

[B58] Dolen G Osterweil E Rao BS Smith GB Auerbach BD Chattarji S Bear MF Correction of fragile X syndrome in mice Neuron 2007 56 955 962 10.1016/j.neuron.2007.12.001 18093519 PMC2199268

[B59] Stanton PK Sarvey JM Blockade of long-term potentiation in rat hippocampal CA1 region by inhibitors of protein synthesis J Neurosci 1984 4 3080 3088 6502226 10.1523/JNEUROSCI.04-12-03080.1984 PMC6564864

[B60] Frey U Krug M Reymann KG Matthies H Anisomycin, an inhibitor of protein synthesis, blocks late phases of LTP phenomena in the hippocampal CA1 region in vitro Brain Res 1988 452 57 65 10.1016/0006-8993(88)90008-X 3401749

[B61] Paradee W Melikian HE Rasmussen DL Kenneson A Conn PJ Warren ST Fragile X mouse: strain effects of knockout phenotype and evidence suggesting deficient amygdala function Neuroscience 1999 94 185 192 10.1016/S0306-4522(99)00285-7 10613508

[B62] Zhang J Hou L Klann E Nelson DL Altered hippocampal synaptic plasticity in the FMR1 gene family knockout mouse models J Neurophysiol 2009 101 2572 2580 19244359 10.1152/jn.90558.2008 PMC2681424

[B63] Abraham WC Williams JM Properties and mechanisms of LTP maintenance Neuroscientist 2003 9 463 474 10.1177/1073858403259119 14678579

[B64] Krug M Lossner B Ott T Anisomycin blocks the late phase of long-term potentiation in the dentate gyrus of freely moving rats Brain Res Bull 1984 13 39 42 10.1016/0361-9230(84)90005-4 6089972

[B65] Nguyen PV Abel T Kandel ER Requirement of a critical period of transcription for induction of a late phase of LTP Science 1994 265 1104 1107 10.1126/science.8066450 8066450

[B66] Kelleher RJ 3rd Govindarajan A Jung HY Kang H Tonegawa S Translational control by MAPK signaling in long-term synaptic plasticity and memory Cell 2004 116 467 479 10.1016/S0092-8674(04)00115-1 15016380

[B67] Cohen AS Abraham WC Facilitation of long-term potentiation by prior activation of metabotropic glutamate receptors J Neurophysiol 1996 76 953 962 8871210 10.1152/jn.1996.76.2.953

[B68] Raymond CR Thompson VL Tate WP Abraham WC Metabotropic glutamate receptors trigger homosynaptic protein synthesis to prolong long-term potentiation J Neurosci 2000 20 969 976 10648701 10.1523/JNEUROSCI.20-03-00969.2000 PMC6774154

[B69] Auerbach BD Bear MF Loss of the fragile X mental retardation protein decouples metabotropic glutamate receptor dependent priming of long-term potentiation from protein synthesis J Neurophysiol 2010 104 1047 1051 10.1152/jn.00449.2010 20554840 PMC2934918

[B70] Lauterborn JC Rex CS Kramar E Chen LY Pandyarajan V Lynch G Gall CM Brain-derived neurotrophic factor rescues synaptic plasticity in a mouse model of fragile X syndrome J Neurosci 2007 27 10685 10694 10.1523/JNEUROSCI.2624-07.2007 17913902 PMC6672822

[B71] Markram H Lubke J Frotscher M Sakmann B Regulation of synaptic efficacy by coincidence of postsynaptic APs and EPSPs Science 1997 275 213 215 10.1126/science.275.5297.213 8985014

[B72] Bi GQ Poo MM Synaptic modifications in cultured hippocampal neurons: dependence on spike timing, synaptic strength, and postsynaptic cell type J Neurosci 1998 18 10464 10472 9852584 10.1523/JNEUROSCI.18-24-10464.1998 PMC6793365

[B73] Desai NS Casimiro TM Gruber SM Vanderklish PW Early postnatal plasticity in neocortex of Fmr1 knockout mice J Neurophysiol 2006 96 1734 1745 10.1152/jn.00221.2006 16823030

[B74] Meredith RM Holmgren CD Weidum M Burnashev N Mansvelder HD Increased threshold for spike-timing-dependent plasticity is caused by unreliable calcium signaling in mice lacking fragile X gene FMR1 Neuron 2007 54 627 638 10.1016/j.neuron.2007.04.028 17521574

[B75] Turrigiano GG The self-tuning neuron: synaptic scaling of excitatory synapses Cell 2008 135 422 435 10.1016/j.cell.2008.10.008 18984155 PMC2834419

[B76] Ibata K Sun Q Turrigiano GG Rapid synaptic scaling induced by changes in postsynaptic firing Neuron 2008 57 819 826 10.1016/j.neuron.2008.02.031 18367083

[B77] Sutton MA Ito HT Cressy P Kempf C Woo JC Schuman EM Miniature neurotransmission stabilizes synaptic function via tonic suppression of local dendritic protein synthesis Cell 2006 125 785 799 10.1016/j.cell.2006.03.040 16713568

[B78] Soden ME Chen L Fragile X protein FMRP is required for homeostatic plasticity and regulation of synaptic strength by retinoic acid J Neurosci 2010 30 16910 16921 10.1523/JNEUROSCI.3660-10.2010 21159962 PMC3073636

[B79] Turrigiano GG Leslie KR Desai NS Rutherford LC Nelson SB Activity-dependent scaling of quantal amplitude in neocortical neurons Nature 1998 391 892 896 10.1038/36103 9495341

[B80] Hu JH Park JM Park S Xiao B Dehoff MH Kim S Hayashi T Schwarz MK Huganir RL Seeburg PH Homeostatic scaling requires group I mGluR activation mediated by Homer1a Neuron 2010 68 1128 1142 10.1016/j.neuron.2010.11.008 21172614 PMC3013614

[B81] Zhong X Li H Chang Q MeCP2 phosphorylation is required for modulating synaptic scaling through mGluR5 J Neurosci 2012 32 12841 12847 10.1523/JNEUROSCI.2784-12.2012 22973007 PMC3474205

[B82] Volk LJ Pfeiffer BE Gibson JR Huber KM Multiple Gq-coupled receptors converge on a common protein synthesis-dependent long-term depression that is affected in fragile X syndrome mental retardation J Neurosci 2007 27 11624 11634 10.1523/JNEUROSCI.2266-07.2007 17959805 PMC6673232

[B83] Connor SA Hoeffer CA Klann E Nguyen PV Fragile X mental retardation protein regulates heterosynaptic plasticity in the hippocampus Learn Mem 2011 18 207 220 10.1101/lm.2043811 21430043 PMC3072772

[B84] Godfraind JM Reyniers E De Boulle K D'Hooge R De Deyn PP Bakker CE Oostra BA Kooy RF Willems PJ Long-term potentiation in the hippocampus of fragile X knockout mice Am J Med Genet 1996 64 246 251 10.1002/(SICI)1096-8628(19960809)64:2<246::AID-AJMG2>3.0.CO;2-S 8844057

[B85] Li J Pelletier MR Perez Velazquez JL Carlen PL Reduced cortical synaptic plasticity and GluR1 expression associated with fragile X mental retardation protein deficiency Mol Cell Neurosci 2002 19 138 151 10.1006/mcne.2001.1085 11860268

[B86] Hayashi ML Rao BS Seo JS Choi HS Dolan BM Choi SY Chattarji S Tonegawa S Inhibition of p21-activated kinase rescues symptoms of fragile X syndrome in mice Proc Natl Acad Sci USA 2007 104 11489 11494 10.1073/pnas.0705003104 17592139 PMC1899186

[B87] Zhao MG Toyoda H Ko SW Ding HK Wu LJ Zhuo M Deficits in trace fear memory and long-term potentiation in a mouse model for fragile X syndrome J Neurosci 2005 25 7385 7392 10.1523/JNEUROSCI.1520-05.2005 16093389 PMC6725289

[B88] Suvrathan A Hoeffer CA Wong H Klann E Chattarji S Characterization and reversal of synaptic defects in the amygdala in a mouse model of fragile X syndrome Proc Natl Acad Sci USA 2010 107 11591 11596 10.1073/pnas.1002262107 20534533 PMC2895119

[B89] Wilson BM Cox CL Absence of metabotropic glutamate receptor-mediated plasticity in the neocortex of fragile X mice Proc Natl Acad Sci USA 2007 104 2454 2459 10.1073/pnas.0610875104 17287348 PMC1892931

[B90] Brown V Jin P Ceman S Darnell JC O'Donnell WT Tenenbaum SA Jin X Feng Y Wilkinson KD Keene JD Microarray identification of FMRP-associated brain mRNAs and altered mRNA translational profiles in fragile X syndrome Cell 2001 107 477 487 10.1016/S0092-8674(01)00568-2 11719188

[B91] Jung H Yoon BC Holt CE Axonal mRNA localization and local protein synthesis in nervous system assembly, maintenance and repair Nat Rev Neurosci 2012 13 308 324 22498899 10.1038/nrn3210 PMC3682205

[B92] Akins MR Berk-Rauch HE Fallon JR Presynaptic translation: stepping out of the postsynaptic shadow Front Neural Circuits 2009 3 17 19915727 10.3389/neuro.04.017.2009 PMC2776480

[B93] Price TJ Melemedjian OK Fragile X mental retardation protein (FMRP) and the spinal sensory system Results Probl Cell Differ 2012 54 41 59 10.1007/978-3-642-21649-7_4 22009347 PMC3213681

[B94] Price TJ Rashid MH Millecamps M Sanoja R Entrena JM Cervero F Decreased nociceptive sensitization in mice lacking the fragile X mental retardation protein: role of mGluR1/5 and mTOR J Neurosci 2007 27 13958 13967 10.1523/JNEUROSCI.4383-07.2007 18094233 PMC2206543

[B95] Davidkova G Carroll RC Characterization of the role of microtubule-associated protein 1B in metabotropic glutamate receptor-mediated endocytosis of AMPA receptors in hippocampus J Neurosci 2007 27 13273 13278 10.1523/JNEUROSCI.3334-07.2007 18045921 PMC6673406

[B96] Lu R Wang H Liang Z Ku L O'Donnell WT Li W Warren ST Feng Y The fragile X protein controls microtubule-associated protein 1B translation and microtubule stability in brain neuron development Proc Natl Acad Sci USA 2004 101 15201 15206 10.1073/pnas.0404995101 15475576 PMC524058

[B97] Darnell JC Jensen KB Jin P Brown V Warren ST Darnell RB Fragile X mental retardation protein targets G quartet mRNAs important for neuronal function Cell 2001 107 489 499 10.1016/S0092-8674(01)00566-9 11719189

[B98] Zalfa F Giorgi M Primerano B Moro A Di Penta A Reis S Oostra B Bagni C The fragile X syndrome protein FMRP associates with BC1 RNA and regulates the translation of specific mRNAs at synapses Cell 2003 112 317 327 10.1016/S0092-8674(03)00079-5 12581522

[B99] Wei ZX Yi YH Sun WW Wang R Su T Bai YJ Liao WP Expression changes of microtubule associated protein 1B in the brain of Fmr1 knockout mice Neurosci Bull 2007 23 203 208 10.1007/s12264-007-0030-1 17687394 PMC5550582

[B100] Chowdhury S Shepherd JD Okuno H Lyford G Petralia RS Plath N Kuhl D Huganir RL Worley PF Arc/Arg3.1 interacts with the endocytic machinery to regulate AMPA receptor trafficking Neuron 2006 52 445 459 10.1016/j.neuron.2006.08.033 17088211 PMC1784006

[B101] Waung MW Pfeiffer BE Nosyreva ED Ronesi JA Huber KM Rapid translation of Arc/Arg3.1 selectively mediates mGluR-dependent LTD through persistent increases in AMPAR endocytosis rate Neuron 2008 59 84 97 10.1016/j.neuron.2008.05.014 18614031 PMC2580055

[B102] Park S Park JM Kim S Kim JA Shepherd JD Smith-Hicks CL Chowdhury S Kaufmann W Kuhl D Ryazanov AG Elongation factor 2 and fragile X mental retardation protein control the dynamic translation of Arc/Arg3.1 essential for mGluR-LTD Neuron 2008 59 70 83 10.1016/j.neuron.2008.05.023 18614030 PMC2743934

[B103] Shepherd JD Bear MF New views of Arc, a master regulator of synaptic plasticity Nat Neurosci 2011 14 279 284 10.1038/nn.2708 21278731 PMC8040377

[B104] Plath N Ohana O Dammermann B Errington ML Schmitz D Gross C Mao X Engelsberg A Mahlke C Welzl H Arc/Arg3.1 is essential for the consolidation of synaptic plasticity and memories Neuron 2006 52 437 444 10.1016/j.neuron.2006.08.024 17088210

[B105] Niere F Wilkerson JR Huber KM Evidence for a fragile X mental retardation protein-mediated translational switch in metabotropic glutamate receptor-triggered Arc translation and long-term depression J Neurosci 2012 32 5924 5936 10.1523/JNEUROSCI.4650-11.2012 22539853 PMC3349238

[B106] Iacoangeli A Rozhdestvensky TS Dolzhanskaya N Tournier B Schutt J Brosius J Denman RB Khandjian EW Kindler S Tiedge H On BC1 RNA and the fragile X mental retardation protein Proc Natl Acad Sci USA 2008 105 734 739 10.1073/pnas.0710991105 18184799 PMC2206605

[B107] Zhang Y Venkitaramani DV Gladding CM Kurup P Molnar E Collingridge GL Lombroso PJ The tyrosine phosphatase STEP mediates AMPA receptor endocytosis after metabotropic glutamate receptor stimulation J Neurosci 2008 28 10561 10566 10.1523/JNEUROSCI.2666-08.2008 18923032 PMC2586105

[B108] Goebel-Goody SM Baum M Paspalas CD Fernandez SM Carty NC Kurup P Lombroso PJ Therapeutic implications for striatal-enriched protein tyrosine phosphatase (STEP) in neuropsychiatric disorders Pharmacol Rev 2012 64 65 87 10.1124/pr.110.003053 22090472 PMC3250079

[B109] Goebel-Goody SM Wilson-Wallis ED Royston S Tagliatela SM Naegele JR Lombroso PJ Genetic manipulation of STEP reverses behavioral abnormalities in a fragile X syndrome mouse model Genes Brain Behav 2012 11 586 600 10.1111/j.1601-183X.2012.00781.x 22405502 PMC3922131

[B110] Westmark CJ Malter JS FMRP mediates mGluR5-dependent translation of amyloid precursor protein PLoS Biol 2007 5 e52 10.1371/journal.pbio.0050052 17298186 PMC1808499

[B111] Westmark CJ Westmark PR O'Riordan KJ Ray BC Hervey CM Salamat MS Abozeid SH Stein KM Stodola LA Tranfaglia M Reversal of fragile X phenotypes by manipulation of AbetaPP/Abeta levels in Fmr1KO mice PLoS One 2011 6 e26549 10.1371/journal.pone.0026549 22046307 PMC3202540

[B112] Nadif Kasri N Nakano-Kobayashi A Van Aelst L Rapid synthesis of the X-linked mental retardation protein OPHN1 mediates mGluR-dependent LTD through interaction with the endocytic machinery Neuron 2011 72 300 315 10.1016/j.neuron.2011.09.001 22017989 PMC3206629

[B113] Muddashetty RS Kelic S Gross C Xu M Bassell GJ Dysregulated metabotropic glutamate receptor-dependent translation of AMPA receptor and postsynaptic density-95 mRNAs at synapses in a mouse model of fragile X syndrome J Neurosci 2007 27 5338 5348 10.1523/JNEUROSCI.0937-07.2007 17507556 PMC6672337

[B114] Todd PK Mack KJ Malter JS The fragile X mental retardation protein is required for type-I metabotropic glutamate receptor-dependent translation of PSD-95 Proc Natl Acad Sci USA 2003 100 14374 14378 10.1073/pnas.2336265100 14614133 PMC283599

[B115] Zalfa F Eleuteri B Dickson KS Mercaldo V De Rubeis S di Penta A Tabolacci E Chiurazzi P Neri G Grant SG Bagni C A new function for the fragile X mental retardation protein in regulation of PSD-95 mRNA stability Nat Neurosci 2007 10 578 587 10.1038/nn1893 17417632 PMC2804293

[B116] Gross C Nakamoto M Yao X Chan CB Yim SY Ye K Warren ST Bassell GJ Excess phosphoinositide 3-kinase subunit synthesis and activity as a novel therapeutic target in fragile X syndrome J Neurosci 2010 30 10624 10638 10.1523/JNEUROSCI.0402-10.2010 20702695 PMC2924772

[B117] Lee HY Jan LY Fragile X syndrome: mechanistic insights and therapeutic avenues regarding the role of potassium channels Curr Opin Neurobiol 2012 22 1 8 10.1016/j.conb.2012.01.004 22483378 PMC3393774

[B118] Lee HY Ge WP Huang W He Y Wang GX Rowson-Baldwin A Smith SJ Jan YN Jan LY Bidirectional regulation of dendritic voltage-gated potassium channels by the fragile X mental retardation protein Neuron 2011 72 630 642 10.1016/j.neuron.2011.09.033 22099464 PMC3433402

[B119] Gross C Yao X Pong DL Jeromin A Bassell GJ Fragile X mental retardation protein regulates protein expression and mRNA translation of the potassium channel Kv4.2 J Neurosci 2011 31 5693 5698 10.1523/JNEUROSCI.6661-10.2011 21490210 PMC3089949

[B120] Strumbos JG Brown MR Kronengold J Polley DB Kaczmarek LK Fragile X mental retardation protein is required for rapid experience-dependent regulation of the potassium channel Kv3.1b J Neurosci 2010 30 10263 10271 10.1523/JNEUROSCI.1125-10.2010 20685971 PMC3485078

[B121] Brown MR Kronengold J Gazula VR Chen Y Strumbos JG Sigworth FJ Navaratnam D Kaczmarek LK Fragile X mental retardation protein controls gating of the sodium-activated potassium channel Slack Nat Neurosci 2010 13 819 821 10.1038/nn.2563 20512134 PMC2893252

[B122] Zhang Y Brown MR Hyland C Chen Y Kronengold J Fleming MR Kohn AB Moroz LL Kaczmarek LK Regulation of neuronal excitability by interaction of fragile x mental retardation protein with slack potassium channels J Neurosci 2012 32 15318 15327 10.1523/JNEUROSCI.2162-12.2012 23115170 PMC3518385

[B123] Chen X Yuan LL Zhao C Birnbaum SG Frick A Jung WE Schwarz TL Sweatt JD Johnston D Deletion of Kv4.2 gene eliminates dendritic A-type K+ current and enhances induction of long-term potentiation in hippocampal CA1 pyramidal neurons J Neurosci 2006 26 12143 12151 10.1523/JNEUROSCI.2667-06.2006 17122039 PMC6675426

[B124] Hoffman DA Magee JC Colbert CM Johnston D K+ channel regulation of signal propagation in dendrites of hippocampal pyramidal neurons Nature 1997 387 869 875 10.1038/43119 9202119

[B125] Watanabe S Hoffman DA Migliore M Johnston D Dendritic K+ channels contribute to spike-timing dependent long-term potentiation in hippocampal pyramidal neurons Proc Natl Acad Sci USA 2002 99 8366 8371 10.1073/pnas.122210599 12048251 PMC123073

[B126] Jung SC Kim J Hoffman DA Rapid, bidirectional remodeling of synaptic NMDA receptor subunit composition by A-type K+ channel activity in hippocampal CA1 pyramidal neurons Neuron 2008 60 657 671 10.1016/j.neuron.2008.08.029 19038222 PMC2637039

[B127] Chuang SC Zhao W Bauchwitz R Yan Q Bianchi R Wong RK Prolonged epileptiform discharges induced by altered group I metabotropic glutamate receptor-mediated synaptic responses in hippocampal slices of a fragile X mouse model J Neurosci 2005 25 8048 8055 10.1523/JNEUROSCI.1777-05.2005 16135762 PMC6725444

[B128] Krueger DD Osterweil EK Chen SP Tye LD Bear MF Cognitive dysfunction and prefrontal synaptic abnormalities in a mouse model of fragile X syndrome Proc Natl Acad Sci USA 2011 108 2587 2592 10.1073/pnas.1013855108 21262808 PMC3038768

[B129] Bolduc FV Bell K Cox H Broadie KS Tully T Excess protein synthesis in Drosophila fragile X mutants impairs long-term memory Nat Neurosci 2008 11 1143 1145 10.1038/nn.2175 18776892 PMC3038669

